# Hospital and Institutional News

**Published:** 1917-06-16

**Authors:** 


### HOSPITAL AND INSTITUTIONAL NEWS.

### OUR HOSPITAL SUNDAY NUMBER.

Next week's issue of The Hospital will be a special Hospital Sunday Number. This year, owing to paper difficulties, we are not publishing our usual Hospital Sunday Supplement, which has been issued regularly each year since 1895, and has been available for distribution for the use of the congregation in every place of worship in the Metropolitan area. The usual statistics, summaries of the year's work in the hospitals and medical charities of London, and particulars of the needs of individual institutions, together with the voluntary hospitals' budget, will, however, appear as part of next week's Special Hospital Sunday Number, which will contain other features of interest to all those concerned in the growth and development of our great hospitals and of the Metropolitan Hospital Sunday Fund.

### SIR ALFRED KEOGH AND SPECIAL HOSPITALS.

At the annual meeting of the South London Hospital for Women, held on Wednesday of last week (June 6), an opinion strongly adverse to the continued existence of special hospitals was expressed by Surgeon-General Sir A. Keogh, Director-General of the Army Medical Service. He said that he saw no necessity for special hospitals for the ears, nose, eyes, throat, heart, and lungs, and he would like to reduce them or abolish them altogether. Not merely on the ground of economy, but on the ground of efficiency also, they should all be welded into the special departments of the great Metropolitan hospitals. Sir A. Keogh drew the line, however, at hospitals for women and children, giving as his reason the fact that in the present state of medical opinion women were excluded from the staffs of the great Metropolitan institutions, "a condition of things which I hope the public will no longer tolerate." He added that it was monstrous that ladies who were qualified and experienced, and who, he claimed, had shown themselves to be as good as men, should be relegated to certain London hospitals, and thus be prevented from doing their duty to the public.

### AMERICAN SURGEONS FOR WORK IN ENGLAND.

When Mr. Balfour went to the United States last month there went with him Colonel T. H. Goodwin, R.A.M.C., who was instructed to ask for the assistance of some American surgeons specially skilled in orthopædic surgery to work in the military hospitals of this country which are devoted to orthopædics. In response to this request, General Gorgas, Surgeon-General of the United States Army, asked Dr. Joel E. Goldthwait, of Boston, and Dr. W. G. Erving, of Washington, to select a number of surgeons, and twenty of them have already started for this country. They have been granted military rank in the United States Army. Their services have been placed at the disposal of Sir Robert Jones, to whom has been given the main share of orthopædic work amongst the British wounded. The demands on the medical profession of the British Isles have been so great, and the number of surgeons here still available is so small, that the assistance of these American surgeons will be certainly of value in dealing with the very many wounded who need special treatment.

### THE VICTIMISATION OF A GENEROUS PROFESSION.

A protest has been raised by a correspondent in the *Morning Post* against the non-payment of performers at war charity concerts, which have been the means of raising large sums of money. "Is it too much to ask," he writes, "that at least a beggarly 10 per cent. out of such profits should be set aside to pay concert artists a bare living wage? . . . I appeal on behalf of those artists whose ordinary concerts and means of livelihood have been killed by these entertainments. No one would dream of protesting against concerts given to wounded soldiers in hospitals. The ordinary war charity concert, however, victimises cruelly a generous profession, and I should like to ask if it is not possible for a few prominent leaders of society to agree among themselves not to lend their names in future to any charity concerts at which the only payment artists receive is 'Thank you for your kind attendance,' even if as much as that." Apart from the statement elsewhere in the letter that "theatre artists can generally afford these gratuitous appearances," which is untrue of the profession as a whole, we welcome the writer's argument. The proper remedy is for the authorities in control of war charities and entertainments on their behalf to insist that a fee of at least one or two guineas should be given to all performers. If the proposal of a percentage were adopted it would only mean that the sum thus derived would be given to some theatrical or musical charity. A set fee would go direct to the performers, who have such a long record of self-sacrifice on behalf of needy charities.

### A HOSPITAL FOR THOSE WHO HAVE LOST LIMBS IN THE WAR.

The Scottish Hospital for Limbless Sailors and Soldiers at Erskine House, near Glasgow, where accommodation is provided for 500 patients, was formally opened by Princess Louise on Wednesday, June 6, in the presence of representatives of the Admiralty and the War Office and a great gathering of the general public. Her Royal Highness, who was presented with an address enclosed in a casket and with an album containing photographs of the hospital, spoke in glowing terms of the beautiful institution and the charm of its surroundings. At Erskine House, she said, they hoped to help brave men to regain much of their former activity and to make life more hopeful to them. Surgeon-General Russell, Deputy-Director of Medical Services, pointed out that the question of limbless men was not a problem merely of to-day or to-morrow. For thirty or forty years there would be thousands of men who would want to come back to that institution as their Alma Mater to have limbs replaced.

### THE HAYES GIFT TO CHESTER INFIRMARY.

Mr. Sneyd Kynnersley, chairman of the board of Chester Royal Infirmary, has announced an important gift to the institution by Mr. and Mrs. G. W. Hayes, who have undertaken to endow a ward of twelve beds in the Albert Wood Wing. For this purpose they have provided a sum of £17,000 in War Loan 5 per Cent. Stock, and wish the ward to be endowed in memory of their son Richard, who died at the Front some months ago. The Chester Royal Infirmary has come through hard times and survived architectural and other difficulties, and we believe that this fine piece of generosity marks the reward which is reaped by all hospitals who admit their deficiencies and boldly try to remedy them as the Chester institution has done.

### AN IRISH HOSPITAL HISTORIAN.

Dr. MacDowel Cosgrave has done well to reprint his historical article on the Drumcondra Hospital from the pages of the Dublin *Journal of Medical Science*, in which it first appeared in December 1916. Round the three principal dates in its history, the opening in 1818 as the Whitworth Fever Hospital, its reopening in 1853 as a general hospital, and its rechristening in 1893 as Drumcondra Hospital, he groups such causes and effects as he can compress into sixteen pages, which conclude with the roll of doctors who have been attached to the institution since its foundation. Its first construction was noteworthy for the cylindrical ventilators which were laid in the floor of each storey and communicated with the open air at one end, and at the other with the inside of the ward by means of valves in the floor. They are still to be seen in the building. It was not till 1842 that any minutes appear to have been kept, so it really had no history for twenty-four years, at the end of which time general cases were admitted. From 1849-50 the hospital was empty. Its reopening was followed by the reception in 1856 of paying patients "at the weekly sum of two shillings." Every patient, however, had to supply his own food at this date. In 1870 the hospital was largely occupied by chronic cases. The first trained nurse to become matron was appointed in 1884, but a proposal to hold an annual meeting, made at the same time, was not even seconded! Though this was soon altered, it was not till 1908 that a constitution and rules were formally prepared. This historical sketch is not the only gift which Dr. Cosgrave has given to the hospital, but it is as valuable as any which it has received, and an epitome of Irish hospital history.

### THE BELFAST EYE HOSPITALS.

Dr. W. M. Killen made an interesting speech at the annual meeting of the Benn Ulster Eye, Ear, and Throat Hospital. He said that it was intended that both the eye hospitals in Belfast should be extended after the war. The Benn Hospital would gain by the addition of fifteen beds, and the fact was that both the Ophthalmic and the Benn Hospitals could be doubled in size to the great advantage of the city. From the financial point of view one had to admit the public subscribed less than one-sixth of the hospital's income, which was a state of affairs that required to be improved. It is pleasant to see two special hospitals working and developing with the harmony that appears to accompany the proceedings of the two ophthalmic hospitals of Belfast. But if extensive additions to either or to both are contemplated, the public must take a far more active and sympathetic interest in them than it does at present, even though they manage to pay their way. In Dr. Killen's analysis of the sources of the hospital's income there is a note of warning which no one contemplating extensions can afford to neglect.

### "A GROWING GRUMBLE" IN SWANSEA.

The monthly meeting of the Swansea Hospital board had to consider the serious question of a growing waiting-list, and of the discontent which is created by it. Mr. T. F. Jones, chairman of the reception committee, reported that there were 200 names upon the waiting-list, and was followed by Mr. G. Gething, who declared that the growing public grumble was increasing. He added that steps were being taken to remedy the difficulty. Mr. Roger Beck, the chairman, stated that all cases were being brought in as quickly as possible, but it is clear that the steps will have to be very definite to meet the difficulty. How far the Swansea hospitals may be able to combine is the important question, since they are all probably faced with the same difficulty, and if the claims of the soldiers are a principal cause in the congestion an effort should be made for more buildings to be taken over in order that the total number of beds available in the district may be increased. The civilian patients must be looked after, and it would be unfair for the civil hospitals to accept so many soldier patients as to keep the waiting-lists of civil patients permanently high. Beds are needed for both, and by a co-operative effort new buildings or temporary wards should be added so that the needs of both classes of patients may be met. Isolated efforts to reduce individual waiting-lists are sure to prove insufficient in the long run.

### HER NATIVE CITY.

Mrs. Georgiana Collett, now of San Francisco, but born in Bristol, has sent 2,000 dollars to the Bristol branch of the Red Cross Society for the purchasing of artificial limbs for crippled soldiers if these are not provided officially. In the latter case she has allotted it for any purpose which the County Director may think fit. Mrs. Collett has also sent 2,000 dollars to the Lord Mayor of Bristol for the relief of soldiers' widows and orphans. Mrs. Collett makes it clear that it is the sentiment which she still feels for her birthplace, as much as her sympathy for the Allied cause, which had prompted her generous gifts to the victims of the war in her native city.

### RIVER TRIPS FOR THE WOUNDED.

WE are glad to see that for the third summer in succession river trips for the wounded have now begun, thanks to the activity of the Red Cross Society and to the generosity of the waterside interests of the Port of London. Saturdays and wet days have been the only ones when the steamers have not made their expeditions up and down the river from Erith to Kew. The men on the first up-river trip, which took place on June 4, were given as a souvenir maps which illustrated the chief places of interest up the river, and tea and cigarettes, of course, formed two of the pleasures of the afternoon. The trips last for four hours, and, on the precedent of last year, number eighty-two in each direction. By their means it is hoped that 14,000 men may be able to enjoy the river this season.

### ST. DUNSTAN'S HOSTEL FOR BLINDED SOLDIERS.

THE report of St. Dunstan's Hostel "for the year ending March 31, 1917," is a record of the considerable industry that has been set in motion by Sir Arthur Pearson and other workers to ensure that everything possible to be done to alleviate the lot of sailors and soldiers blinded in the war is done now, and, in regard to the future of these men, that provision is made to ensure that slackening of interest on the part of the public, who have up to now showed their keen sympathy by means of handsome pecuniary support, cannot materially affect the welfare of our blind heroes. Up to the present, according to a statement made by Mr. Barnes in the House of Commons, 600 men have been blinded in the war; 210 of these have passed through St. Dunstan's, 300 are there now, and 100 will enter when they are ready. The Government did a very wise thing in entrusting this piece of work to one institution and making it clear that no further agency was required. The result has been an entire absence of overlapping, and the work has been very well done indeed. One excellent thing is that the men are not spoiled; from the first the idea is impressed upon them that they are to continue to be useful members of society, and that by special training the burden of blindness is made not to rest too heavily upon the energies of the worker.

### HOSPITAL SUPPLIES OF WINES AND SPIRITS.

THE consumption of wines and spirits varies greatly amongst voluntary hospitals—for example, in one year the cost under this heading was more at the Great Northern Central Hospital than at St. Thomas's Hospital, where there are three or four times as many beds. The extent of the consumption depends entirely on the opinion of the medical men in charge of the cases, and in most of the smaller hospitals, if wines and spirits are used at all, what is required is easily obtained at a local shop. It is presumably in the interests of the larger hospitals that the British Hospitals Association has made inquiry of the Advisory Committee of Customs and Excise, it having been discovered that hospitals requiring large quantities of wines and spirits were unable to obtain them through the usual channels. The answer given is that the Advisory Committee will consider applications and may grant additional authorities for the clearance of wines and spirits. Applications should be addressed to the Secretary of the Advisory Committee, Customs and Excise, 110 Cannon Street, E.C. 4, setting forth particulars of wines and spirits used in 1916, the source of supply then, and the name and address of the firm from whom present supplies will be bought. Fortunately this latest addition to the cares of the caterer affects only a very few institutions, and even in these the use of wines and spirits, except in respect of a very few patients, can easily be dispensed with.

### A NEW RED CROSS HOSPITAL AT MONMOUTH.

LADY LLANGATTOCK, in opening a new Red Cross hospital at Monmouth, remarked that, as a member of the committee responsible for looking after and entertaining overseas nurses, she had had many interesting conversations with those from Australia and Canada, and they all expressed astonishment at the immense amount of good work done by the women of England. As president of the Monmouthshire Red Cross Society Lady Llangattock said she could not imagine any hospital better adapted for its work than the new Red Cross hospital at Monmouth, and thanks were due to the honorary secretary, Mrs. Crompton-Roberts, and the commandant, Mrs. W. H. Williams. Miss Batchelor is matron of the new hospital.

### THE VALUE OF SMALL HOSPITALS.

ALTHOUGH it cannot be denied that small hospitals are expensive in proportion to the amount of work they can do, so that the cost per patient is decidedly greater than in larger hospitals, yet it is hardly necessary to mention that something can be said on the other side. General Sir Alfred Keogh, Director-General of the Army Medical Service, recently opened a small hospital in Portland Place which has been furnished and fitted by Mr. S. J. Waring, by whom also it will be maintained. It is intended to serve as an annexe to Queen Alexandra's Hospital for Officers at Highgate. Sir Alfred Keogh acknowledged that small hospitals were expensive, but he maintained that it was imperative to retain them at present, and that to do away with them ruthlessly would be little short of a catastrophe. The atmosphere in a small hospital is frequently more homelike than that of a large institution, and this is perhaps especially appreciated by officers. At all events, whatever may be thought of the question from an economical point of view, no one who knows the facts can deny that the small private hospitals of this country have done invaluable work in this national crisis.

### A HOSPITAL'S FRIENDS.

THE Nottingham Children's Hospital, which has been treating the children of soldiers, we understand, on special terms, has lately paid off a debt of over £2,000 by means of the issue of a private appeal by two members of the board. They appealed to some of the hospital's friends of their acquaintance, and the debt was more than met in this private fashion. Happy is the institution which collects round it an inner circle of friends, for though this form of private appeal is not one to which resort can always be had, in hard times it is something to fall back on, and does not interfere with the next general appeal which may be required.

### THE OLD SITE OF NEWCASTLE INFIRMARY.

The Estate and Property Committee of the Newcastle Corporation has recommended the sale of the western part of the old infirmary site, comprising an area of 3,155 square yards, to the Co-operative Wholesale Society. The Sanitary Committee has since reported that if this sale were made it would prevent the erection of a public abattoir, a scheme for which has been actively canvassed from July 1916. The Sanitary Committee believes that such a scheme would give to Newcastle a pre-eminent position in the North as a distributing centre, and unrivalled means of importing and storing frozen and chilled meat and other foods from abroad. If it were adopted, the cattle market, it is proposed, should be moved to a part of the site which would include that which has been recommended for sale to the Co-operative Society, where 2,000 cattle could be accommodated. By this means the site of the present cattle market could be used for building a public abattoir. The price suggested is £6 per square yard. It will be interesting to follow the developments of these proposals: old sites of hospitals often prove to be contentious ground.

### PANEL PATIENTS WITHOUT A DOCTOR.

Should a panel doctor be expected to attend, without charge, a person who states that he or she is insured but who does not produce documentary evidence of that fact? The Birmingham Insurance Committee answered the question in the negative last week, when it rejected by eighteen votes to eleven a proposal of the Medical Service Sub-Committee that a doctor be asked to refund a charge of 7s. made in such circumstances. Dr. H. G. Dain, who moved the amendment, took exception to the statement that some doctors were in the habit of getting fees from insured persons. He urged that it was unfair to expect medical men to provide treatment because a man said he was entitled to it, without offering any evidence. Dr. F. A. L. Burges added that the medical profession would never accept the ruling that they were to attend any person who simply said he was insured. Such a policy would result in chaos, because insured persons would not then take the trouble to select their panel doctors at all. Whether the decision here given will survive an appeal remains to be seen. It is certainly a matter which should be authoritatively settled.

### THE NEWEST WAR HOSPITAL.

Bournemouth Corporation has provided a new war hospital. The building is that which has been the Park Prewett Asylum. The main asylum and the three villas are now officially occupied and in the hands of the Canadian military authorities, under the command of Colonel Roberts and his staff. The male sick and infirm block is already furnished with beds and furniture for hospital use. The theatre at present is used as a temporary store for unpacking goods. The stewards' store is now in use as a military pack store. Upholsterers' and tailors' shops have been altered in construction, and transformed into an operating theatre, anæsthetic and $x$-ray rooms. The plumbers' shop is now a general store. The nurses' home is equipped ready for the matron and nursing sisters. An unloading platform is under construction, the whole length of the stewards' store, and on a siding of the through line of railway. Colonel Willan visited the asylum and spent some hours going round the works, at the same time meeting Colonel Roberts. The heating of the main building has now been on for some time; official tests have been made, and all is satisfactory. The bakery is officially in the military hands, with a corporal baker and two privates working it; the laundry is also in use when required. The kitchen is occupied by the Canadians, and is used as kitchen and mess-room. The large bath-houses have been regularly in use for some time.

### THE DIMINUTION OF PANEL DOCTORS IN BIRMINGHAM.

The number of Birmingham panel doctors on military service is sixty, and when new arrangements which are now being carried into effect are completed the total will be further increased, and then the city will be left with only nine practitioners of military age. In order to meet the difficulties of this situation the Birmingham Insurance Committee proposes to establish a central surgery, which will be opened without delay. If this proves a success, more surgeries may be established. This is a new departure, and the working of the scheme will be watched with much interest.

### A CRECHE WARD IN MANCHESTER.

The Manchester Corporation has sanctioned the establishment of a crèche ward at the Monsall Hospital, and it is now suggested that greater use should be made of it in the way of providing treatment for young children suffering from wasting, malnutrition, and diarrhœa. It is estimated that the maintenance of ten young children will amount to £3 15s. a week, and that the nursing will cost £1 8s. 6d. a week. It is hoped that the Local Government Board will include the scheme under child welfare work. Of the utility of such a scheme there can be no doubt, for it is just in such cases as these that institutional treatment can be of the greatest service.

### THE NEED FOR DAY NURSERIES.

Dr. Francis J. Allan, the medical officer of health for Westminster, commenting in his annual report for last year upon the declining birth-rate, says that whereas the death-rate of legitimate children was 68 per 1,000, that of illegitimate children was as high as 230 per 1,000. He observes that there is evidence that attempts have been made to procure abortion in a number of cases both among married and unmarried women, and that serious illness has in several cases resulted to the mothers. Many married women are now absolutely compelled to go out to work, and the need for day nurseries has been much felt. There are two in Westminster, but as the women frequently go to work before the nurseries open in the morning they have not always been able to avail themselves of them. The provision of nurseries where the children could be left from Monday to Saturday has been suggested, and they would at the same time give health visitors in training at the nurseries valuable experience. The need for proper homes for illegitimate children is also pointed out in the report.

### DIRTY WORKERS.

THE liberty of the subject appears to be in jeopardy by reason of a novel suggestion brought forward by the Manchester Corporation Sanitary Committee at the instance of the medical officer of health. The medical officer has had correspondence with the Home Office Chief Inspector of Factories with regard to compelling persons who are verminous to submit to medical examination, because of the danger to the health of the district caused by the existence of such dirty persons. The Sanitary Committee suggest that the Home Office and Local Government Board should be asked to bring forward legislation enabling local authorities to exclude from work all dirty people until they are certified to be free from vermin.

### THE RISK OF INFECTION FROM DENTAL INSTRUMENTS.

MANY cases are on record which demonstrate clearly that unless due care be taken the extraction of a tooth entails a risk of infection by venereal disease. A sub-committee of the Health Committee of the South Shields Corporation reports that it has been considering instances of cases of persons running the risk of contracting disease at the hands of unqualified dental practitioners who are in the habit of extracting teeth without taking the necessary care that the instruments used are thoroughly sterilised. Therefore the Health Committee has drawn the attention of the Local Government Board to the risk of infection by venereal disease through unqualified dental practitioners, and suggested that steps should be taken to suppress the practice of these persons. The danger certainly exists, and is fully recognised by all those dental practitioners who are qualified, but it is ignored by many who practise without any qualification, and often with very little training.

### THE DEARTH OF DENTAL SURGEONS.

THE serious position of dental surgery was again brought to the notice of the Southwark Tribunal by Dr. S. M. Pembery, when he asked for exemption until the end of November for nine dental students at Guy's Hospital, who by that time will have sat for their final examinations. He pointed out that at Guy's they had only been able to attend to the needs of 10,748 out-patients in the dental department from January 1 this year to the end of May, whereas in 1914 the patients numbered during the same period 17,939. This was not due to the lack of patients, for many could not be treated owing to the shortage of dentists, and he had to tell the Tribunal that if these nine students went they would have to close the dental department altogether. Chairs and tables were covered over at the hospital because of the great lack of dentists. Moreover, if the students went into the Army without completing their full course they would simply be privates, but if they were fully qualified they would be able to take commissions probably in the R.A.M.C. Many English dentists were privates in the Forces, whilst American and Colonial dentists were being brought to this country to do the very necessary work amongst the wounded soldiers. Naturally the Tribunal realised the seriousness of the situation, for they granted the students exemption until they had completed their course of training at the hospital, and had sat for their final examinations.

### THE LATE DR. BEZLY THORNE.

ON the eve of its Annual General Meeting the Royal British Nurses' Association has, through the death of Dr. Bezly Thorne, sustained a loss which will be deeply regretted throughout the Corporation. Its members must feel that they have not only lost a Chairman who has given long years of generous and devoted service, but that, individually, each has lost a friend, for he has shown for many years that the interests of the Nursing Profession were very close to his heart. A little incident serves to indicate that it was in this light that the members of the R.B.N.A. had come to regard the Chairman who, with quiet dignity, has presided at their meetings for so many years. Shortly after the announcement of his death, among many messages of regret, a request reached the office of the Association from a number of nurses that they might be accorded the privilege of choosing the flowers to be sent from the Corporation as a token of esteem and affection. The efforts of Dr. Bezly Thorne to promote the welfare of the Association have been unwearying, and it seems fitting that the last public act in his beneficent career was done on its behalf when, by request of Her Royal Highness the Princess Christian, he occupied the chair at the historic meeting of the Corporation in January last. To quote his own words on that occasion, he "was wrapped up in the Association," and we have only to look back across the years from the days when he acted as Honorary Secretary and to those later twenty years when he held the Vice-Chairmanship to understand with what complete confidence the members of the Association learnt to seek his counsel and to follow it gladly. We offer to the members of the R.B.N.A. and to its President our sincere sympathy in the loss they have experienced by the sudden death of Dr. Bezly Thorne.

### REMOVAL OF THE BOARD OF EDUCATION.

THERE can be no doubt that the various departments of the Government show great energy in expanding and in requiring new premises. The latest demand has been for the removal of the Board of Education from their present offices in order to accommodate the War Staff. This change necessitates the provision of fresh premises for the Board of Education, and the Victoria and Albert Museum at South Kensington has been requisitioned for the purpose. The curtailment of the museum facilities is greatly to be regretted, though it is hoped that it will not be found necessary to close the museum entirely. The closure of other museums in London has already reduced greatly the opportunities of the public and students, and we trust that no further seizures of museums will be considered necessary.

### THE DEARTH OF RECRUITS FOR THE V.A.D.

At present we are told that there is no waiting list for the Voluntary Aid Detachment. Several hundred nursing members of the V.A.D. are wanted to go to Salonica, yet they cannot be obtained. There are several grievances which need recognition and redress, and we doubt if the supply of recruits will increase until some improvement is made. One grievance is that the unpaid V.A.D. has to pay her own railway fare at the full rate, for she does not benefit by any of the concessions which are granted to many other war workers. This grievance is especially felt by those who have farthest to travel, as when the home is in Scotland or Ireland. Further, the unpaid V.A.D. has to pay the cost of her medical examination, which is half a guinea or a guinea, while candidates for the Women's Auxiliary Corps for France are examined free. Moreover, an unpaid V.A.D. has to provide her own uniform, and it is said that in some hospitals she has to pay for her board. There are several other grievances, some of more importance than others, which at least need careful investigation in the near future, if the supply of nurses from this source is to be maintained. All who have worked with V.A.D.s will bear willing witness to their zeal, their energy, and their keenness in their work; so it is a great pity that any real grievance should go unredressed.

### CYCLES FOR DOCTORS AND NURSES.

Many local authorities are providing health visitors with bicycles, and at Southampton the County Council reports that it would be advantageous for the assistant county medical officers to possess motor-cycles. Already these officers are required to keep cycles, but as the cost of running a motor-cycle appears to be about 3d. a mile, there is no inducement to them to procure motor-cycles, although it would obviously be of advantage to the county service that they should do so, especially during the present curtailment of railway services and increase of fares. In the circumstances it is suggested that 3d. a mile shall be paid as travelling allowance, so that the doctors need no longer hesitate to purchase motor-cycles.

### POOR-LAW VISITORS.

Better times are in store for Poor-Law women visitors. Birmingham Guardians have considered the scale of salary, which now commences at £75 per annum, rising by annual increments of £5 to a maximum of £100 a year. Having regard to the salaries paid in other large towns, and also to the health visitors in the service of the Birmingham Corporation, the Guardians have raised the scale to £80, rising to £110 per annum. At the same time, the Board has given the present staff a £5 increase.

### MENTAL HOSPITAL ATTENDANTS AND THE TRIBUNALS.

The conditional-exemption certificates of forty-one members of the staff of the County Mental Hospital were contested by the military representative at the Lancaster Tribunal. Although it was pointed out that half the patients were now being nursed by women, and that further to reduce the male staff would be prejudicial to administration and the public, six men in Class A were taken. The matter is serious since mental patients are concentrated in the institutions which have not been taken over as military hospitals, and entirely to staff the mental hospitals by women would be a mistake, especially when skill and training are required before any man or woman can be given responsibility for the nursing of such patients.

### THE EX-PATIENT AND HOSPITAL GAZETTES.

The *Searchlight*, the magazine of the 2nd Western Hospital, Manchester, still manages to keep in touch with some of its former staff and patients, from whom correspondence and contributions are received from different parts of the world. Illustrations and verse occur to diversify the pages, and Goethe is quoted in England's praise. The quotation, like others of its class, seems to show how vain it is to attempt to sum up a nation in a paragraph. There is also an article on "Culture," which shows that an attempt is made to keep the "Bookman's Corner" up to date. If it were possible to enlarge the space devoted to contributions from former members of the hospital it would be a welcome addition, but no form of contributions is harder to secure.

### THIS WEEK'S DRUG MARKET.

A slight improvement that was noticeable in business towards the end of last week has barely been maintained, and a distinctly quiet tone now pervades the market. At existing high prices people are naturally disinclined to purchase more than the smallest quantities that will suffice to carry them over the immediate future. Quantities of some drugs that used to cost so many shillings now cost almost as many pounds, and under the circumstances it is quite an easy matter to run up a big bill with very little to show for it. The upward movement in the prices of salicylic acid and sodium salicylate continues, but the rise has been gradual, without any sharp upward movements. Quinine is quiet, with very little change in prices. The tendency of the price of peppermint oil is inclined upwards, but business is quiet. The large quantities of honey that are offered have had the effect of depressing prices, which, however, are still extraordinarily high. Cape aloes is rather dearer. Cardamoms are slightly lower in price. The value of ipecacuanha is well maintained. Opium still tends upwards in price, but the quotation for morphine is unchanged.

